# The Clusters-in-a-Liquid Approach for Solvation: New Insights from the Conformer Specific Gas Phase Spectroscopy and Vibrational Optical Activity Spectroscopy

**DOI:** 10.3389/fchem.2016.00009

**Published:** 2016-02-25

**Authors:** Angelo S. Perera, Javix Thomas, Mohammad R. Poopari, Yunjie Xu

**Affiliations:** Department of Chemistry, University of AlbertaEdmonton, AB, Canada

**Keywords:** solvent effects, induced solvent chirality, vibrational circular dichroism, Raman optical activity, rotational spectroscopy, conformer specific spectroscopy, solute-water H-bonding interaction, explicit and implicit solvation

## Abstract

Vibrational optical activity spectroscopies, namely vibrational circular dichroism (VCD) and Raman optical activity (ROA), have been emerged in the past decade as powerful spectroscopic tools for stereochemical information of a wide range of chiral compounds in solution directly. More recently, their applications in unveiling solvent effects, especially those associated with water solvent, have been explored. In this review article, we first select a few examples to demonstrate the unique sensitivity of VCD spectral signatures to both bulk solvent effects and explicit hydrogen-bonding interactions in solution. Second, we discuss the induced solvent chirality, or chiral transfer, VCD spectral features observed in the water bending band region in detail. From these chirality transfer spectral data, the related conformer specific gas phase spectroscopic studies of small chiral hydration clusters, and the associated matrix isolation VCD experiments of hydrogen-bonded complexes in cold rare gas matrices, a general picture of solvation in aqueous solution emerges. In such an aqueous solution, some small chiral hydration clusters, rather than the chiral solutes themselves, are the dominant species and are the ones that contribute mainly to the experimentally observed VCD features. We then review a series of VCD studies of amino acids and their derivatives in aqueous solution under different pHs to emphasize the importance of the inclusion of the bulk solvent effects. These experimental data and the associated theoretical analyses are the foundation for the proposed “clusters-in-a-liquid” approach to account for solvent effects effectively. We present several approaches to identify and build such representative chiral hydration clusters. Recent studies which applied molecular dynamics simulations and the subsequent snapshot averaging approach to generate the ROA, VCD, electronic CD, and optical rotatory dispersion spectra are also reviewed. Challenges associated with the molecular dynamics snapshot approach are discussed and the successes of the seemingly random “*ad hoc* explicit solvation” reported before are also explained. To further test and improve the “clusters-in-a-liquid” model in practice, future work in terms of conformer specific gas phase spectroscopy of sequential solvation of a chiral solute, matrix isolation VCD measurements of small chiral hydration clusters, and more sophisticated models for the bulk solvent effects would be highly valuable.

## Introduction

Water is an essential component for life on Earth and provides the necessary environment where most of the biologically important processes take place. It has been recognized for some time that water is not a simple bystander but rather an active participant in these biological events. For example, water is not only required to maintain enzymes in their natural conformation to deliver their full functionality but it can also participate directly in enzymatically catalyzed reactions (Rezaei et al., 2007). Increasingly, researchers are pushing for a detailed and accurate description of solute-water interactions at the molecular level, even in much more complex biological systems (Hummer and Tokmakoff, 2014).

For more than two decades, spectroscopists working in the field of cluster science have been using the bottom-up approach to probe solute-water hydrogen (H)-bonding interactions in great detail. The general idea is that by adding water molecules one at a time, one may gain significant insight into the first few steps of solvation and ultimately “bridge” the gap between the behavior of an isolated solute to that in aqueous solution. For example, Wester and co-workers demonstrated that interaction with just one water molecule is enough to affect the outcome of the nucleophilic substitution reaction of CH_3_I + OH^−^ (Otto et al., 2012), highlighting the significance of such explicit H-bonding interaction. Spectroscopists have used isomer and sometimes even conformer specific spectroscopic techniques to provide direct, bond-specific information about local solute-water interactions in the gas phase. For example, using the conformer selective double resonance laser spectroscopy such as IR–UV ion dip and UV–IR and UV–UV hole burning methods, Simons and co-workers found that while gas phase monosaccharides may prefer somewhat different conformations, interaction with just a single water molecule was enough to lock all of them into the same type of conformation as in water (Çarçabal et al., 2005; Drouin et al., 2009). As highlighted by Cocinero and Çarçabal in their recent review, the first bound water molecule plays the role of a “conformational lock” in these cases by replacing the weakest intramolecular H-bond interaction with two strong intermolecular H-bonds and strengthening the H-bond cooperativity effects (Cocinero and Çarçabal, 2015). Pate and co-workers reported a rotational spectroscopic study of sequential solvation of β-propiolactone with one to five water molecules using a chirped pulse Fourier transform microwave (CP-FTMW) spectrometer and revealed the associated microsolvation structures of water surrounding the simple organic molecule (Pérez et al., 2015a,b). Johnson and his team have published a series of studies on vibrational spectral signatures of the proton defects in the 3D protonated (water)_21_ clusters and other smaller protonated water clusters (Fournier et al., 2014, 2015). These results have significantly advanced our knowledge of solute-water interactions at the microscopic scale, and tested the current most accurate theoretical description of solute-waster interaction potentials and pushed further theoretical developments.

In aqueous solution and at interfaces with water, new advances in experimental techniques and theoretical treatments make it possible to tease out signatures of solute-water interactions which were previously masked by the bulk water signal. Some noticeable spectroscopic techniques used include ultrafast femtosecond visible, infrared, and terahertz spectroscopies, nonlinear interfacial spectroscopies, electron and nuclear magnetic resonance, and X-ray and neutron spectroscopy and scattering. For example, using two-dimensional infrared and transient absorption spectroscopy, Tokmakoff and co-workers characterized the vibrational couplings by examining the response in the whole mid-infrared region after exciting the OH stretching transition (Ramasesha et al., 2013). Such studies provide insight into the ability of liquid water to dissipate energy through ultrafast vibrational relaxations. This in turn can strongly influence chemical reaction outcomes and stability of reactive intermediates in aqueous solution. Panman et al. found “lubrication” effect of water in H-bonded molecular machines using time-resolved vibrational spectroscopy and NMR line shape analysis (Panman et al., 2013). For water in biological systems, a review article in 2011 (Zhong et al., 2011) and a recent special issue in the Journal of Chemical Physics titled “Biological Water” nicely summarized some recent progresses (Hummer and Tokmakoff, 2014). In addition, the molecular thermodynamics community has been developing new approaches to model thermodynamic properties of water (Dufal et al., 2015). All these research activities have advanced our understanding about various roles water plays in aqueous solution significantly.

Given that the research activities in the gas phase and in the condense phase are often carried out by scientists in very different sub-disciplines, it is not surprising that questions have been raised about what the highly precise gas phase data can provide in terms of understanding the properties of a solute in aqueous solution. Geissler (2013) pointed out in a recent review article titled “Water Interfaces, Solvation and Spectroscopy” that “*Spectroscopic tools that enable the precise characterization of small molecules and clusters in the gas phase are much less directly informative when applied to the fluctuating molecular environment of a liquid. … It can be tempting to reconstruct such spectra as linear combinations of idealized resonances, each notionally corresponding to a distinct class of related configurations; unfortunately, there is usually little basis for doing so*.” Indeed, the dynamic nature of the water solvent emphasized by time-resolved spectroscopic techniques have generated some doubts about the possible direct links from properties of small hydration clusters to those obtained in aqueous solution.

Our research group and others have utilized vibrational optical activity (VOA) spectroscopy, namely vibrational circular dichroism (VCD) and Raman optical activity (ROA), to probe solvent effects on VOA spectral features in the past 10 years. As will be demonstrated in this review, extensive experimental and theoretical chiroptical studies show that VCD and ROA spectroscopies are highly sensitive to water solvent effects which include both bulk water solvent effects and explicit H-bonding interactions between a chiral solute and water molecules directly in aqueous solution (Sadlej et al., 2006, 2010; Yang and Xu, 2008, 2011; Gobi et al., 2011) Most importantly, unique VCD spectral features have been detected at the water bending band region, i.e., in the so-called chirality transfer spectral window (Losada and Xu, 2007; Losada et al., 2008a,b; Yang and Xu, 2009), indicative of the explicit H-bonding interactions mentioned above. Further, VCD studies of amino acids and their derivatives in water (Zhu et al., 2012; Poopari et al., 2012a, 2013b) have confirmed the importance of including bulk water environment in simulating solvent effects in conjunction with the explicit H-bonding interactions. Built on the foundation of the aforementioned experimental data and the related theoretical investigations, we have proposed a “clusters-in-a-liquid” approach to account for water solvent effects in chiroptical spectra effectively. In addition, the corresponding high resolution spectroscopic studies of the related small chiral hydration clusters will also be presented. The chiroptical spectral features, especially VCD features, of small chiral hydration clusters and of those obtained in aqueous solution will be examined in detail to identify the connection between them.

The remainder of this review is organized as follows. After a brief review of VCD and ROA spectroscopy (Section Brief Overview of VCD and ROA Spectroscopy), we will use two examples to demonstrate the exceptional sensitivity of VCD spectroscopy to bulk solvent effects and to intermolecular H-bonding interactions in solution (Section VCD Sensitivity to Solvent Effects). The main part of this review will deal with the applications of VCD and ROA spectroscopy to water solvation effects in aqueous solution (Section Water Solvation). Most significantly, we will focus on chirality transfer from a chiral solute to water, also termed “induced solvent chirality,” and how these signatures allow one to identify the relatively “long-lived” small hydration clusters in aqueous solution (Section Unique VCD Spectral Signature of Induced Water Chirality in Aqueous Solution). The gas phase rotational spectroscopic studies of small chiral hydration clusters will be discussed in terms of their predicted VCD spectral features and the possible links to those obtained in solution (Section The Small Gas Phase Hydration Clusters and Matrix Isolation VCD Studies). Additional experimental support from the matrix isolation VCD studies of H-bonded chiral complexes in cold rare gas matrices will also be included. In Section The Importance of Bulk Solvent Surrounding, we will examine the importance of bulk solvent environment. The recent ROA studies of water solvation effects, especially those using molecular dynamics simulations, are described in Section ROA Spectroscopy of Chiral Molecules in Water. In Section Clusters-in-a-Liquid Approach, we summarize the “clusters-in-a-liquid” approach using a few examples and discuss the pros and cons of other approaches based mainly on molecular dynamics (MD). Finally, we offer concluding remarks and future outlook of this important research topic.

## Brief overview of VCD and ROA spectroscopy

VCD is the differential absorbance of left vs. right circularly polarized light by a chiral molecule accompanying a vibrational transition. The ratio of VCD vs. IR intensity is usually in the range of 10^−6^−10^−4^. In the last two decades, VCD spectroscopy has experienced significant developments in both experimental and theoretical aspects (Freedman et al., 2003; Stephens et al., 2008; Polavarapu, 2012). Some noticeable improvements in VCD instrumentation have been reported (Malon and Keiderling, 1988, 1996, 1997; Polavarapu, 1989; Nafie, 2000; Lakhani et al., 2009). It is now possible to obtain excellent quality VCD spectra with a commercial FT-VCD spectrometer offered by a number of companies including BioTools and Bruker. Density functional theory (DFT) calculations of VCD signs and intensities have been implemented into many popular electronic structure packages such as Gaussian suite of programs (Frisch et al., 2010) and Amsterdam DF (ADF)^1^. VCD has emerged in the last decade as a powerful new spectroscopic tool for steoreochemical information, such as the absolute configurations of a wide range of chiral compounds in solution. VCD offers several advantages over other more traditional techniques such as X-ray crystallography, NMR shift agent approach such as Mosher Ester method (Dale and Mosher, 1973; Seco et al., 2004; Hoye et al., 2007), total synthesis route, and electronic CD (ECD). First, VCD experiments can be performed directly in solution without the need of a crystal or additional synthetic steps. Second, as an optical spectroscopic method, it can register signals from individual conformers in contrast to X-ray and NMR which have intrinsically slow response to structural changes. Third, VCD spectra show distinct spectral responses from different functional groups with much narrower bandwidth in comparison with ECD, allowing a more conclusive assignment. Finally, VCD calculations involve only the ground electronic state and are quite reliable in comparison to calculations of ECD and optical rotatory dispersion where information about the excited electronic states is needed. Besides its main application for steoreochemical information of chiral compounds, VCD has been recognized in the past few years as a decisive spectroscopic tool to probe inter- and intramolecular H-bonding interactions directly in solution. Of significant interest here is its power in revealing previously unavailable information about the H-bonding topology of water molecules which are directly H-bonded to a chiral solute in aqueous solution.

The second VOA technique called ROA refers to the chiral version of a regular Raman scattering. It measures either the small circularly polarized component generated by a chiral sample in a regular Raman scattered radiation, or more commonly the intensity difference of Raman scattering for right vs. left circularly polarized incident light by a chiral compound. Generally, the ratio of ROA to Raman scattering is in the order of 10^−4^−10^−2^, making ROA an extremely weak process since Raman itself is already a weak process. Hug, Barron, and co-workers had developed some of the most sensitive and versatile ROA instruments in the world (Hug and Hangartner, 1999; Hug, 2003). Hug's latest design was adopted in the current commercial ROA spectrometer offered by BioTools which is capable of routine ROA measurements with high quality. Parallel to VCD theoretical development, DFT calculations of ROA signs and intensities have been implemented into many commercial electronic structure packages, although the calculations are slightly more involved than VCD. While ROA spectroscopy has been widely applied to studies of biological systems such as sugars and proteins (Barron et al., 2007; Barron and Buckingham, 2010), we will only focus on studies related to solvent effects in aqueous solution here.

## VCD sensitivity to solvent effects

VCD spectral features demonstrate not only high sensitivity to bulk solvent effects but also to explicit H-bonding interactions in solution. Below we use two examples to showcase these two main advantages of VCD spectroscopy for solvation studies.

**Figure 1:**
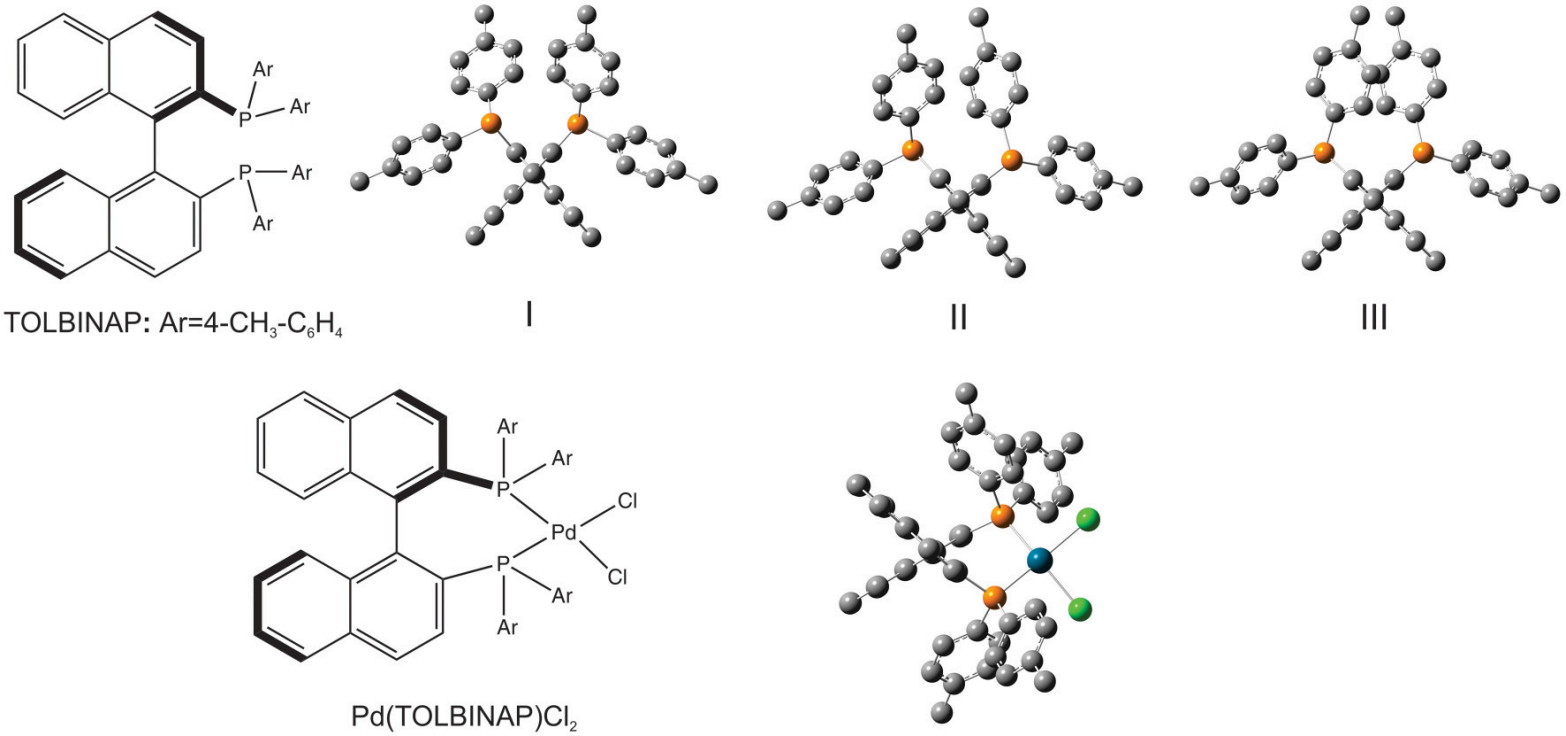
**Chemical formula of the ligand, TOLBINAP, and its Pd complex**. Geometries of the three TOLBINAP conformers, I, II, and III, and the only dominant conformer of Pd(TOLBIANP)Cl_2_ are also given. Reproduced with permission from Dezhahang et al. (2012). Copyright 2012, The Royal Society of Chemistry.

**Figure 2:**
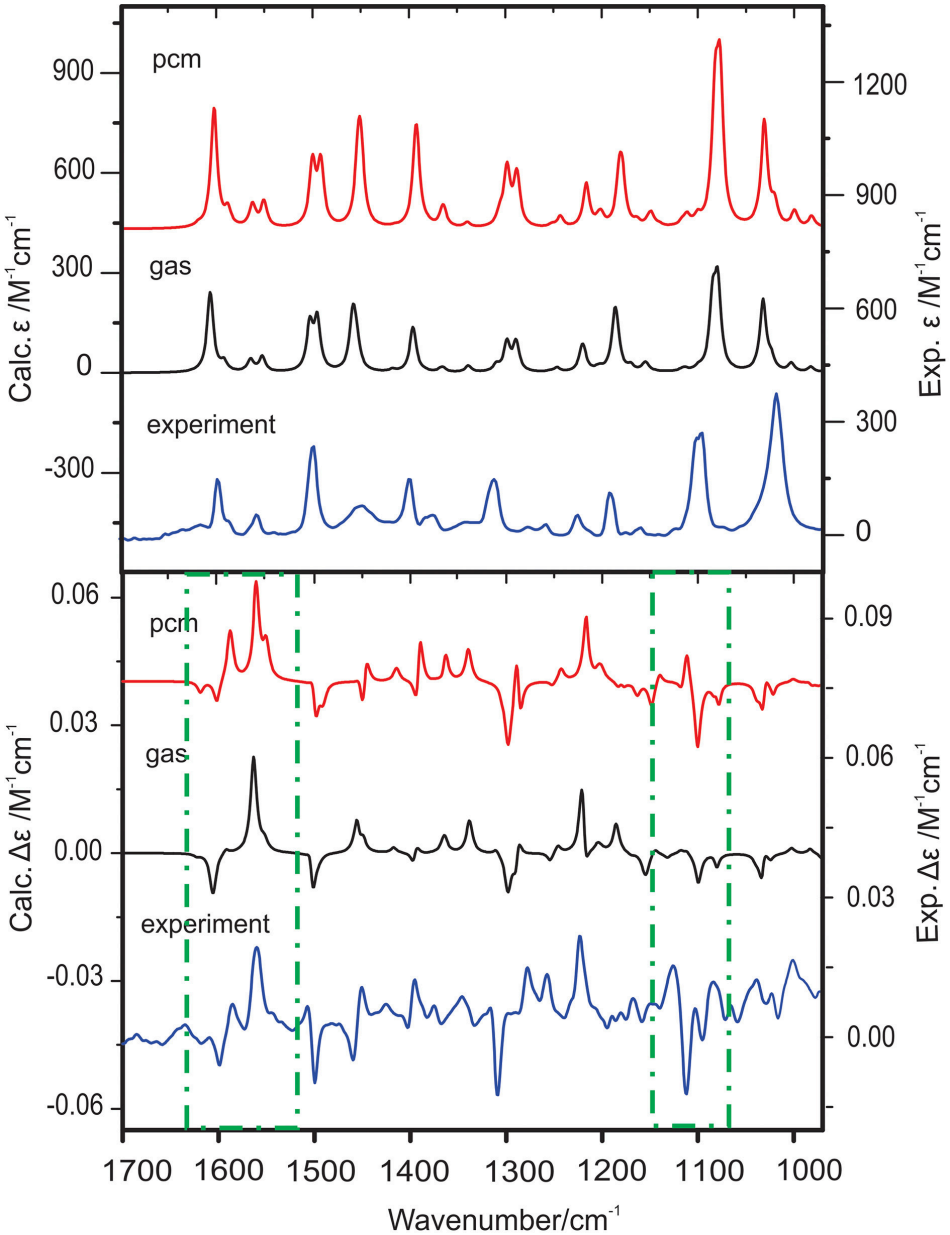
**Comparison of the simulated IR and VCD spectra in the gas phase and with PCM for CDCl_**3**_ with the corresponding experimental spectra of Pd(TOLBINAP)Cl_**2**_ in CDCl_**3**_**. The regions showing noticeable different VCD patterns in the gas phase and in solution are highlighted with dashed-dotted squares. Reproduced with permission from Dezhahang et al. (2012). Copyright 2012, The Royal Society of Chemistry.

A VCD study of two chiral binaphthyl diphosphine ligands and their palladium complexes in solution was reported by Dezhahang et al. (2012) These two ligands are BINAP (2,2′-diphenylphosphino−1,1′-binaphthyl) and its derivative TOLBINAP (Figure 1). While TOLBINAP exists in three different conformations, relating to the different relative orientations of the aromatic rings: parallel, slip parallel, and perpendicular (or face to edge), coordination to Pd restricts the motions of the aromatic rings and results in a single conformation (Figure 1). Comparison of the experimental IR spectrum of the Pd complex measured in CDCl_3_ with the related gas phase simulation is given in Figure 2. While the usual IR frequency shift between the experiment in solution and the calculation in the gas phase is obviously there, it is straightforward to correlate the predicted and the experimental IR bands. The situation, however, is different in terms of VCD where noticeable spectral pattern discrepancies are seen in the regions around 1600 and 1100 cm^−1^. To better account for the bulk solvent effects of CDCl_3_, the implicit solvent model, i.e., integral equation formalism polarizable continuum model (IEF-PCM) (Tomasi and Persico, 1994; Cramer and Truhlar, 1999; Tomasi et al., 2005), was utilized. The resulting IR spectrum looks essentially the same as the one obtained in the gas phase although with a slightly higher overall intensity. The PCM VCD spectrum, on the other hand, is clearly different from the gas phase one, and shows much better agreement in the 1600 cm^−1^ and the 1100 cm^−1^ regions with the experimental data than the gas phase simulation. Since the Pd(TOLBINAP)Cl_2_ complex has only one pretty rigid conformer, the structural variation from the gas phase to solution is quite subtle. This example demonstrates the high sensitivity of VCD spectral features to bulk solvent effects when compared to IR spectral features.

**Figure 3:**
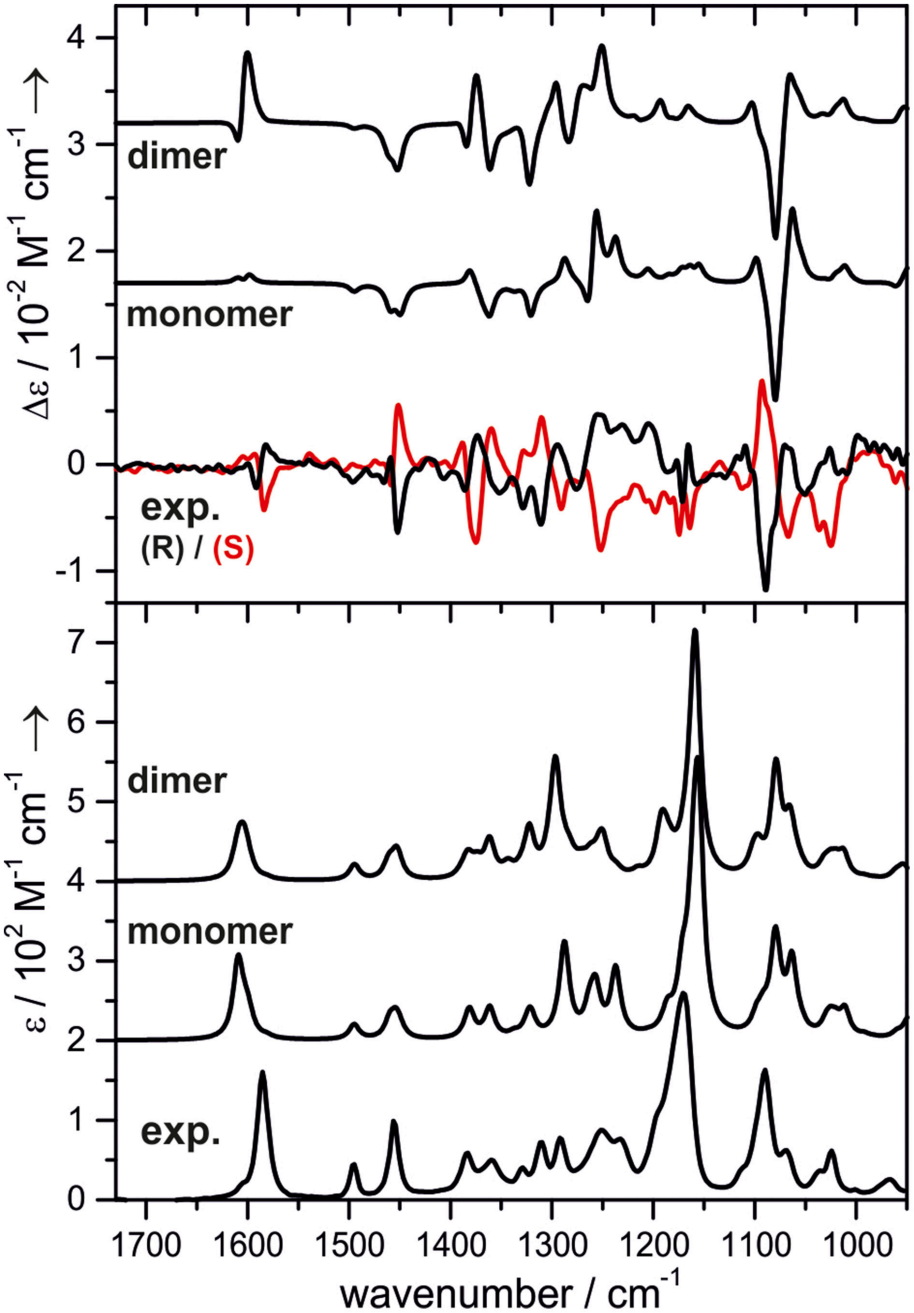
**Experimental and calculated VCD (top) and IR spectra (bottom) of methylbenzyl-amine-borane**. The calculated spectra are offset for clarity. Reproduced with permission from Merten et al. (2014a,b). Copyright 2014, Wiley-VCH Verlag GmbH & Co. KGaA, Weinheim.

**Figure 4:**
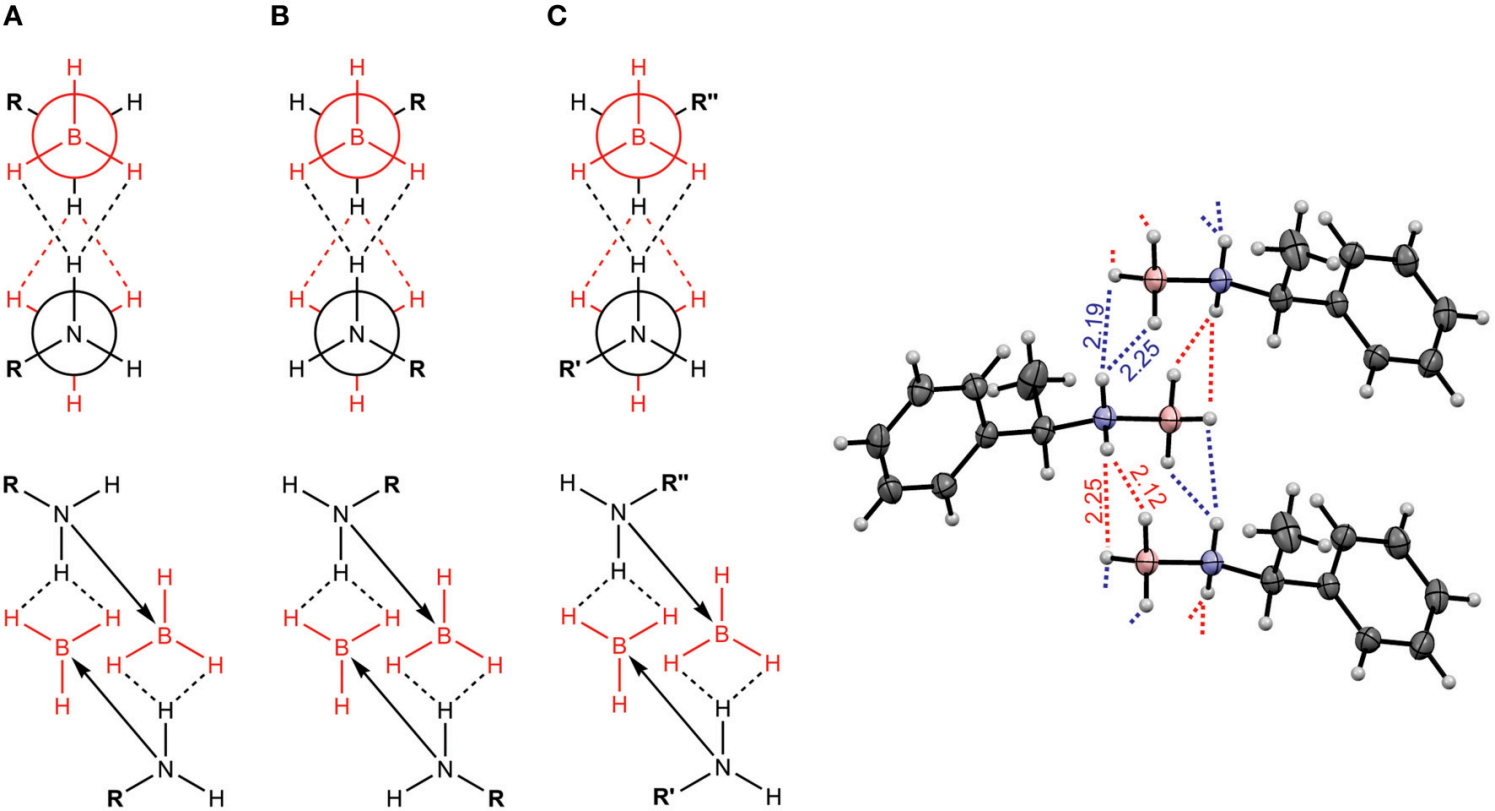
**Three possible binding topologies, (A–C), for the methyl-benzyl-amide borane dimer (left) and the crystal structure of the DHB dimer (right)**. Reproduced with permission from Merten et al. (2014a,b). Copyright 2014, Wiley-VCH Verlag GmbH & Co. KGaA, Weinheim.

**Figure 5:**
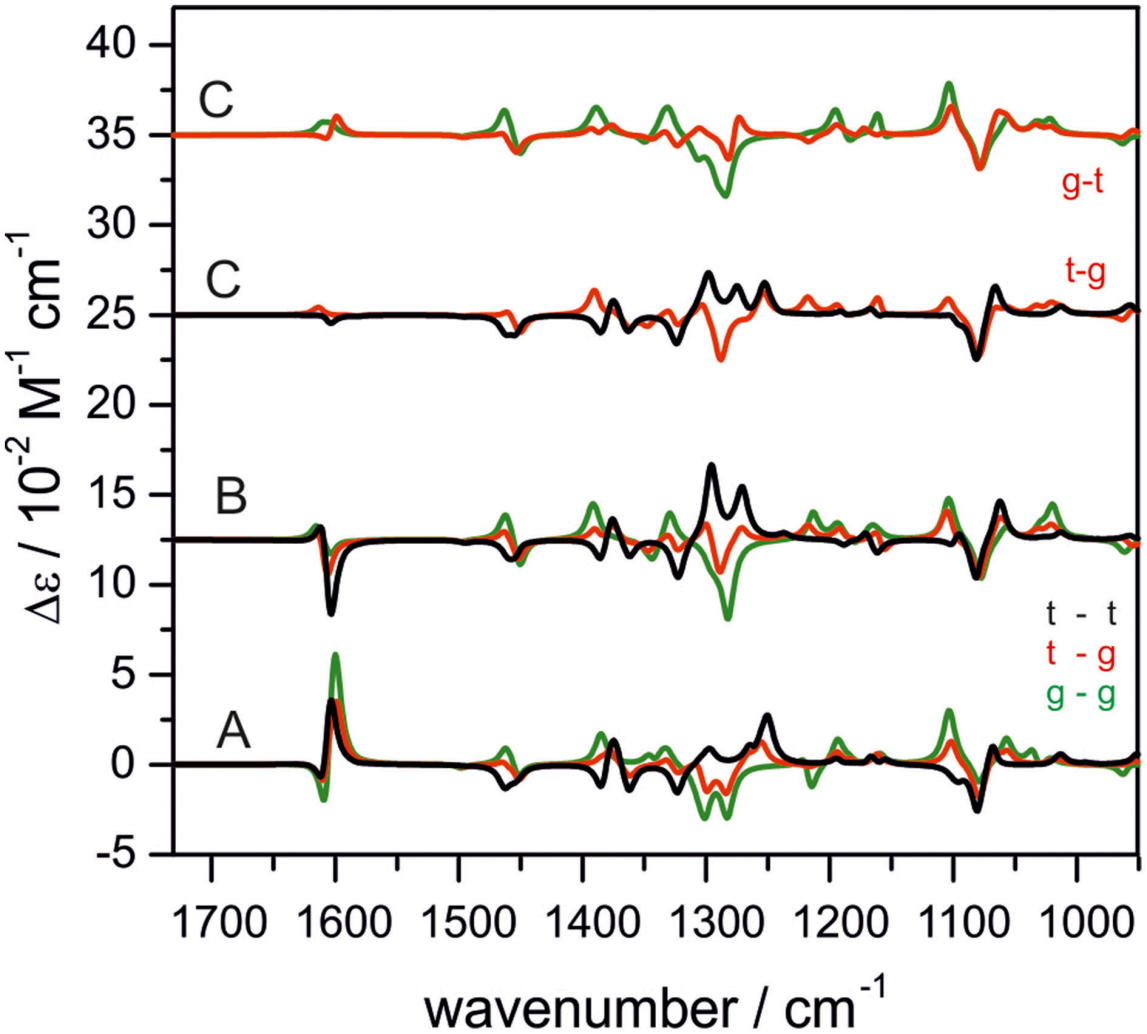
**The calculated single conformer VCD spectra of the dimer of methyl-benzyl-amide borane adopting three binding topologies shown in Figure 4**. Reproduced with permission from Merten et al. (2014a,b). Copyright 2014, Wiley-VCH Verlag GmbH & Co. KGaA, Weinheim.

One research focus of our group is to utilize VCD spectroscopy to probe H-bonding interactions directly in solution. An interesting system studied is a chiral amine borane, namely methylbenzyl-amine-borane (Merten et al., 2014a,b). Ammonia boranes have attracted much interest as potential hydrogen storage material. The simplest ammonia borane, H_3_N·BH_3_, is a white solid at room temperature with a melting point of 113°C, in contrast to −181.3°C of ethane which is isoelectronic (Staubitz et al., 2010). A special kind of H-bond, named dihydrogen bond (DHB): N-H^δ+···δ−^H-B, is identified to be responsible for its unusual stability (Richardson et al., 1995). While the crystal structure study proves that such DHBs exist in the methylbenzyl-amine-borane crystal, one question before the study was whether such DHBs could survive in solution. Figure 3 compares the experimental IR and VCD spectra of methylbenzyl-amine-borane in CDCl_3_ with the calculated ones of the monomer and the DHB dimer (Merten et al., 2014a,b). While some minor changes in IR features are noted going from the monomer to dimer, it is difficult to draw any concrete conclusion from the experimental data about the existence of DHBs, i.e., the existence of dimer, in solution. The VCD features, in particular the +∕− VCD couplet of the NH_2_ bending vibration at ~1585 cm^−1^ and the well-resolved −∕+∕− pattern in the 1400–1350 cm^−1^ region arising from the CH and CH_3_ bending vibrations can only be reproduced by VCD features of the DHB dimer. The chiral amide borane may adopt three different DHB binding topologies between the two monomeric subunits as shown in Figure 4. The corresponding single conformer VCD spectra are summarized in Figure 5. As one can see, only binding topology A offers agreement between the solution VCD and the theoretical data. This observation indicates while the crystal structure takes on binding topology C (Figure 4), in solution the dominant DHB binding topology changes to A. This example not only demonstrates the unique VCD sensitivity to H-bonding interactions in solution but also shows that the dominant structure adopted by the chiral dimer in solution differs from that in the solid state.

The above two examples showcase the sensitivity of VCD spectroscopy to both bulk solvent effects and explicit H-bonding interactions directly in solution. The noticeable advantages of VCD spectroscopy over IR spectroscopy in clearly identifying the bulk solvent effects and the specific intermolecular H-bonding interactions are demonstrated. In the following, we discuss the utility of VCD spectroscopy for probing explicit H-bonding interactions between a chiral solute and water molecules directly in aqueous solution.

## Water solvation

Evaluation of water solvent effects is significantly more complicated than for other protic solvents because water has a strong tendency to form extensive H-bonding networks in aqueous solution. Another major challenge is the very dynamic nature of water solvent. A range of experimental spectroscopic techniques, such as NMR and femtosecond time-resolved Stokes shift, have been used to capture dynamical properties of protein-bound water. The mean residence time (MRT), which measures how long water molecules stay near a biomolecule, was reported using NMR to be in the order of 10 ns–10 ms for interior water and 300–500 ps for surface-bound water (Otting et al., 1991), although subsequent studies reported a much smaller value of 30–50 ps (Denisov and Halle, 1996; Grebenkov et al., 2009). The discrepancy was attributed to the “contamination” from the very slow water in interior to the average water time scale since the NMR method is not site specific (Saha et al., 2015). Using femtosecond spectroscopy, Zhong and co-workers reported a detailed study of tryptophan solvation dynamics in bulk water with femtosecond resolution where an even shorter solvation time of 7~20 ps was obtained (Lu et al., 2004). It remained unclear, however, whether some tightly bound clusters consisting of a chiral solute and a few water molecules are the dominant species in aqueous solution and are responsible for the observed chiroptical signatures in solution or if some dynamical treatment of a chiral solute itself in aqueous solution is essential to recover the experimental spectrum.

One main question is: are there any concrete pieces of evidence for such relatively long-lived chiral hydration clusters in aqueous solution? Based on the above example of the H-bonded solute dimer, we hypothesized that if unique VCD spectral features which are exclusively associated with such chiral hydration clusters could be observed reliably with similar band width as VCD bands in other aprotic solvents such as CCl_4_, then one can confirm the existence of such dominant hydration clusters. In general, the solvation picture at the molecular level appears highly complicated, making it challenging to propose an efficient way to account for water solvent effects. In the following, we present the foundation for our “clusters-in-a-liquid” model where the dominant explicit chiral solute-water H-bonded clusters identified are placed in a bulk water environment modeled by, for example, PCM.

### Unique VCD spectral signatures of induced water chirality in aqueous solution

In 1996, for the first time, Jalkanen et al. attempted to include the explicit H-bonding interaction between water molecules and N-acetyl-L-alanine N'-methylamide (L-AANMA) in their interpretation of the observed VCD spectrum (Jalkanen and Suhai, 1996). They optimized geometries of eight low energy conformers of L-AANMA in the gas phase and solvated one conformer with four additional water molecules, and calculated their IR and VCD spectra. The authors commented that adding water molecules has a significant effect on the appearance of VCD spectrum. Only a few VCD studies concerning water solvent effects had been reported before 2007 (Sadlej et al., 2010). Since 2007, Xu and co-workers have reported a series of VCD studies of prototype chiral molecules such as propylene oxide (PO) (Losada et al., 2008a), methyl lactate (ML) (Losada and Xu, 2007), lactic acid (Losada et al., 2008b), and glycidol in water (Yang and Xu, 2009). The main experimental data and conclusions are summarized below.

**Figure 6:**
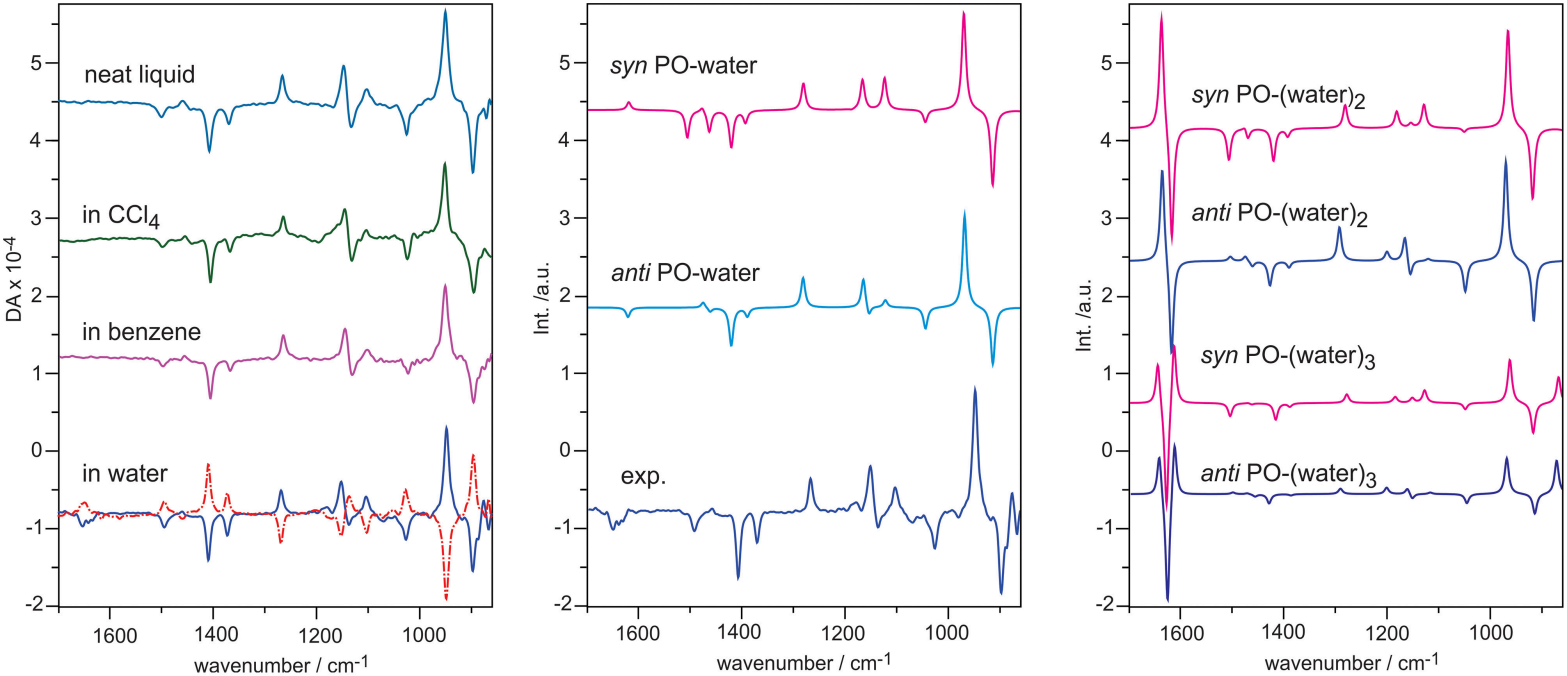
**Left: experimental VCD spectra of ***S***-PO as neat liquid, in CCl_**4**_, in benzene, and in water**. Dotted line indicates measurement with *R*-PO. **Middle**: comparison of the experimental VCD of *S*-PO in water with the simulated VCD spectra of the *anti* and *syn* PO-water complex. **Right**: simulated VCD spectra of the two most stable conformers of PO-(water)_2, 3_. Reproduced with permission from Losada et al. (2008a). Copyright 2008, American Chemical Society.

PO is a simple chiral molecule with a rigid structure except the internal rotation of the methyl group. It is also a commonly used benchmark chiral molecule in the development of theoretical description of VCD. Figure 6 shows the experimental VCD spectra of *S*-PO in a number of solvents such as benzene, CCl_4_, water, and as neat liquid (Losada et al., 2008a). While VCD spectral features in all traces look largely the same, there is a very noticeable additional negative VCD feature at the water bending region for PO in water. To be sure that this is not due to artifacts, VCD measurement was also carried out with the opposite enantiomer. A good mirror-imaged spectrum was obtained for all VCD bands including the one observed at the water bending region. Since water is an achiral molecule, one obvious question is how achiral water molecules gain VCD intensity. In such a solution, some water molecules are likely explicitly H-bonded to a chiral PO. Since water is a part of the chiral H-bonded complex(es), it is natural for the water subunit to show VCD signatures, just as any other functional groups in the complex(es). This phenomena is known as chirality transfer or induced solvent chirality.

How does one model such an induced solvent chirality feature? We first considered the 1:1 PO-water complex where water can approach PO either from the same side as the methyl group (*syn*) or from the opposite side (*anti*). The single conformer VCD spectra of the 1:1 complex are also given in Figure 6. It is immediately obvious that the VCD water bending band is highly sensitive to how water approaches PO. The VCD water bending band is positive for the *syn* orientation and negative for the *anti* one. In addition, there are also changes in the rest of the frequency region. Clearly, *anti* PO-water should be the dominant species in order to achieve a good agreement with the experimental data. How about further explicit interactions with additional water molecules? The simulated VCD spectra of PO-(water)_2,3_ in the *anti* and *syn* positions are presented in Figure 6. If these larger hydration clusters were the main species, one would expect to see significantly enhanced VCD signatures at the water bending region. Obviously, this is not what was observed experimentally. One can therefore draw the conclusion that in such an aqueous solution, the 1:1 PO-water complex is the dominant species which contributes significantly to the observed VCD spectrum. Furthermore, *anti* is the preferred orientation in comparison to *syn*. This conclusion is also supported by the ORD study reported in the same publication (Losada et al., 2008a). In another ORD study carried out using MD simulations, Beratan and co-workers (Mukhopadhyay et al., 2006) took out 10,000 snapshots of the 1:1 PO-water complex in the *anti* and *syn* configurations and calculated their ORD values. The authors found while those corresponding to the *syn* configurations sum up roughly to zero, those related to *anti* sum up to a positive value, in agreement with the experimental data. For the 1:2 PO-(water)_2_ complex, the ORD values sum up to a close to zero value for both *syn* and *anti* configurations. The authors therefore concluded that the main species contributing the ORD experimental value in such an aqueous solution is the 1:1 PO-water complex and *anti* is the preferred orientation, consistent with the conclusion drawn using VCD data.

Overall, the observation of the medium strength VCD water bending band with similar bandwidth as in other solvents is a strong indication that the dominant species responsible for the observed VCD spectrum are the hydration clusters rather than PO itself. Furthermore, we emphasize that while the 1:1 PO-water complex is the dominant species in water, this does not preclude the existence of other larger hydration clusters in such aqueous solution. In Losada et al. (2008a), empirically 6% 1:2 and 4% 1:3 PO-water complexes were included to reproduce the experimental data in solution. In addition, for the 1:1 PO-water complex, the ratio is 74:26% for *anti* vs. *syn*.

**Figure 7:**
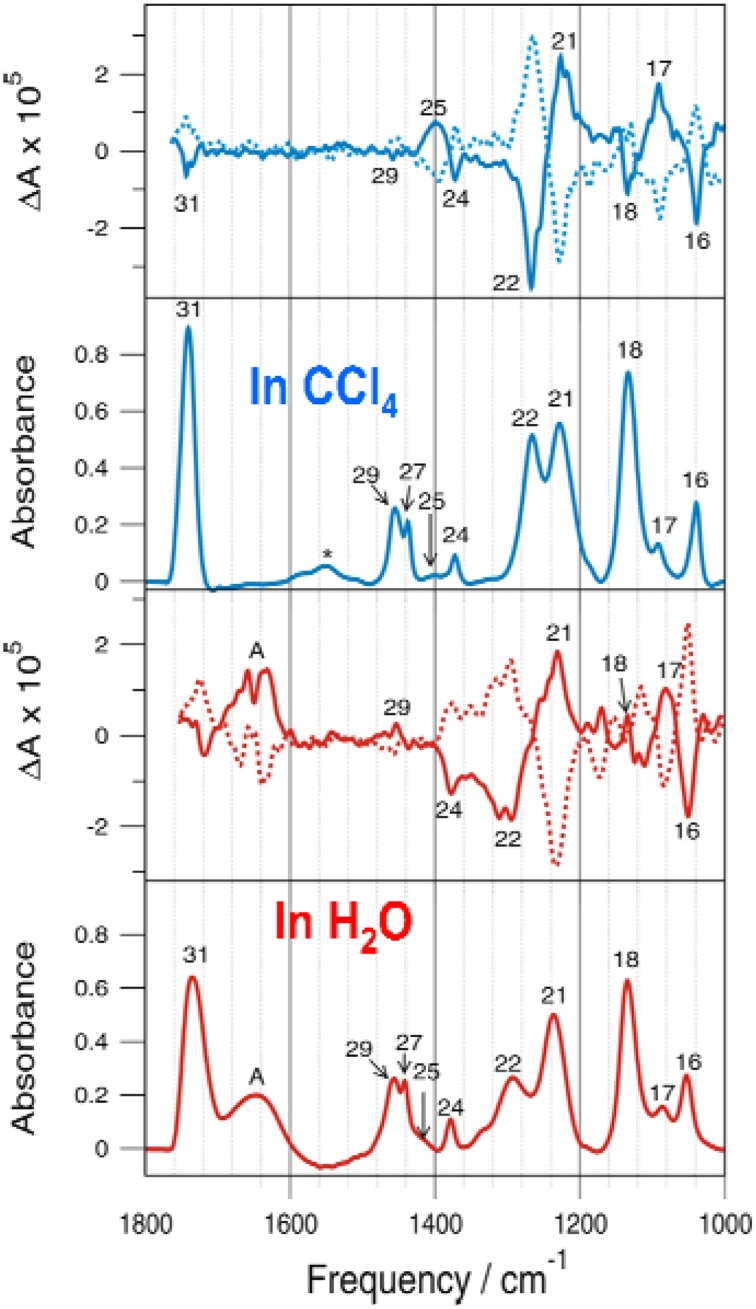
**Comparison of the experimental IR and VCD spectra obtained for ***S***-ML in CCl_**4**_ and in water**. For the VCD section, the spectra of *R*-ML are shown in dotted line. Reprinted with permission from Losada and Xu (2007). Copyright 2007, The Royal Society of Chemistry.

Another system investigated is ML. In Figure 7, the experimental IR and VCD spectra in CCl_4_ and in water are compared. Again, we see clear VCD spectral features at the water bending region at ~1640 cm^−1^. Good mirror-imaged relationships were obtained with *S*- and *R*-ML in CCl_4_ and in water for all VCD bands including the water bending bands, as anticipated (Losada and Xu, 2007). From a simplistic point of view, the more complex VCD features imply that there are likely more than one water molecules which are explicitly H-bonded to the ML molecule or water likes to attach at different sites.

Similarly, VCD spectra of lactic acid (Losada et al., 2008b) and glycidol (Yang and Xu, 2009) also show unique VCD chirality transfer features at the water bending region. The strength and the bandwidth of the induced solvent VCD spectral features at the water bending region support the proposal that these small hydration clusters are the dominant species in water. In the analyses of the solution VCD chirality transfer features, it appears that water prefers specific binding sites and orientations in aqueous solution. The latter observation seemed somewhat unusual, considering the very dynamic nature of water solvent. Therefore, in the following, we present the conformer specific spectroscopic studies of some of these small hydration clusters in a jet environment using high resolution spectroscopy. We then examine whether the main conformer(s) identified in the gas phase bear any connection to those in aqueous solution by comparing their simulated IR and VCD spectra to the related spectra obtained in aqueous solution.

### The small gas phase hydration clusters and matrix isolation VCD studies

**Figure 8:**
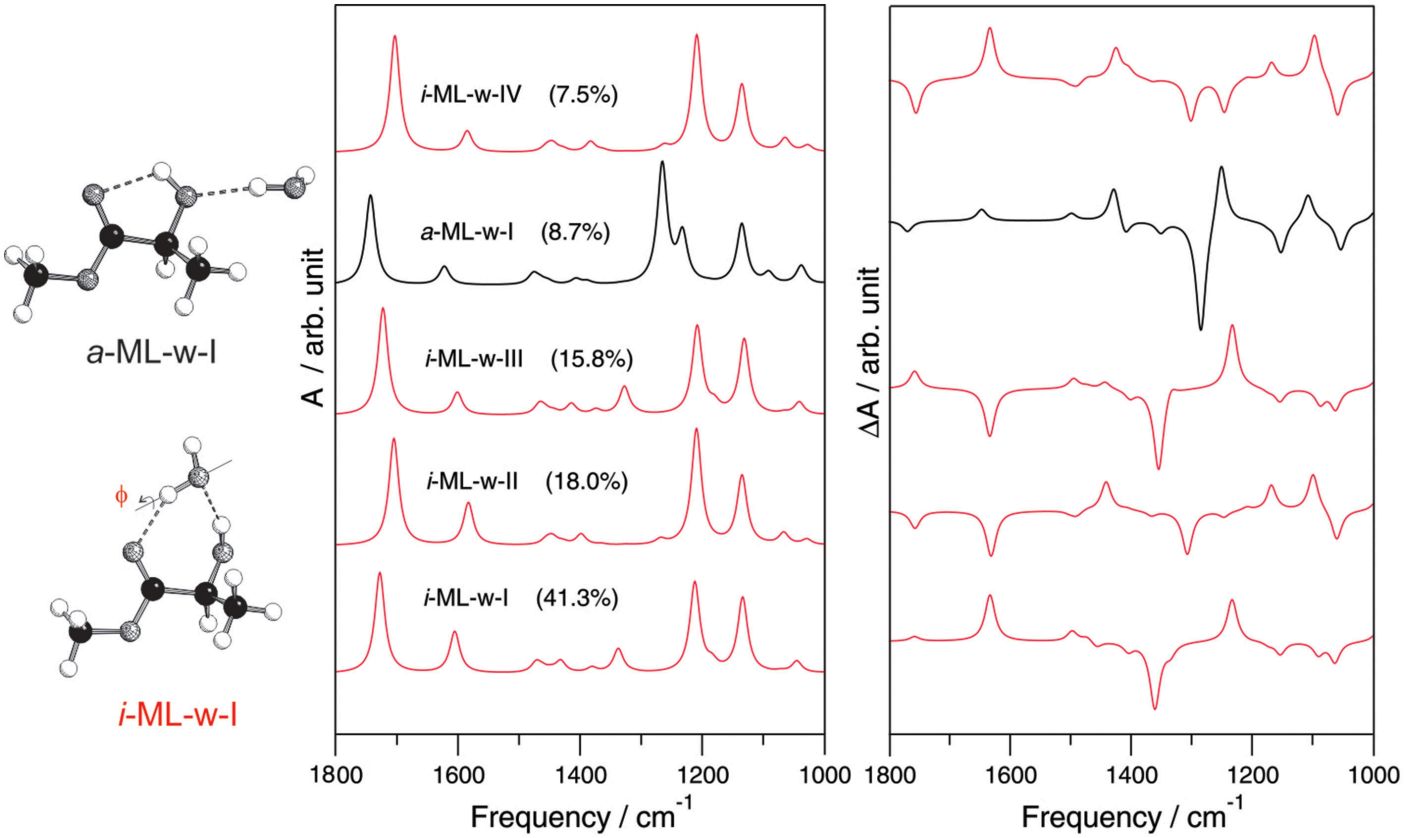
**The simulated IR and VCD spectra of the five most stable conformers of the 1:1 ML-water complex**. The *insertion* and *addition* binding topologies of the ML-water complex are shown on the left. Reprinted with permission from Losada and Xu (2007). Copyright 2007, The Royal Society of Chemistry.

Broadband CP-FTMW and cavity based FTMW spectroscopies were used to probe the unique pointing directions of the free OH bonds in the ML-water and ML-(water)_2_ complexes (Thomas et al., 2014a,b). ML is an α-hydroxy ester with multiple functional groups, i.e., three H-bond acceptor sites and one H-bond donor site. For the ML itself, the only conformer detected in a jet expansion is stabilized by an intramolecular five membered H-bonded ring including the OH and C=O groups (Ottaviani et al., 2006; Borho and Xu, 2007). Two general binding topologies, *insertion* and *addition*, are shown in Figure 8, where water is inserted into the existing intramolecular H-bond of ML or attached to the hydroxy O, leaving the intramolecular H-bond intact, respectively. For the 1:1 complex, four *insertion* conformers look similar, except with different Φ angle values, reflecting the fact that either electron lone pairs of hydroxyl O or carbonyl O can be involved in forming the H-bonds. Three *addition* conformers were predicted. In the case of the ML-(water)_2_ complex, a total of 16 ternary conformers were identified. All the lowest energy conformers are *insertion* only, whereas *insertion*-*addition* or *addition* only conformers are less stable.

For the 1:1 complex, only the most stable ML-water conformer predicted was detected experimentally. For the 1:2 complex, the experimentally observed conformer was not the most stable one predicted at the MP2/6-311++G(d,p) level, rather the second most stable conformer predicted. This reflects some deficiency in the current ability to predict relative energy ordering correctly and emphasizes the need for experimental spectroscopic data in order to unambiguously identify the conformational preference in the gas phase (Xu and Jäger, 2011). In both the 1:1 and 1:2 complexes of ML with water, a strong preference for the *insertion* binding topology over the *addition* binding topology was observed. One interesting and somewhat unexpected observation is the strong preference for a particular orientation of the non-bonded OH group(s) of water in these two hydration clusters. How do the subtly different *insertion* conformations influence the associated IR and VCD spectral features? In Figure 8, simulated IR and VCD spectra of the five most stable 1:1 ML-water complex are compared. The IR spectra of all four *insertion* conformers are quite similar, but differ from that of the *addition* conformer, reflecting their different H-bonding sites. The corresponding VCD spectra, on the other hand, differ drastically even among the four *insertion* conformers, especially in the chirality transfer window where opposite VCD signs are predicted. We note that the most stable 1:1 conformer provides the correct sign for the induced solvent chirality features observed in aqueous solution. Indeed, the main species identified in solution is the same one identified in the gas phase.

**Figure 9:**
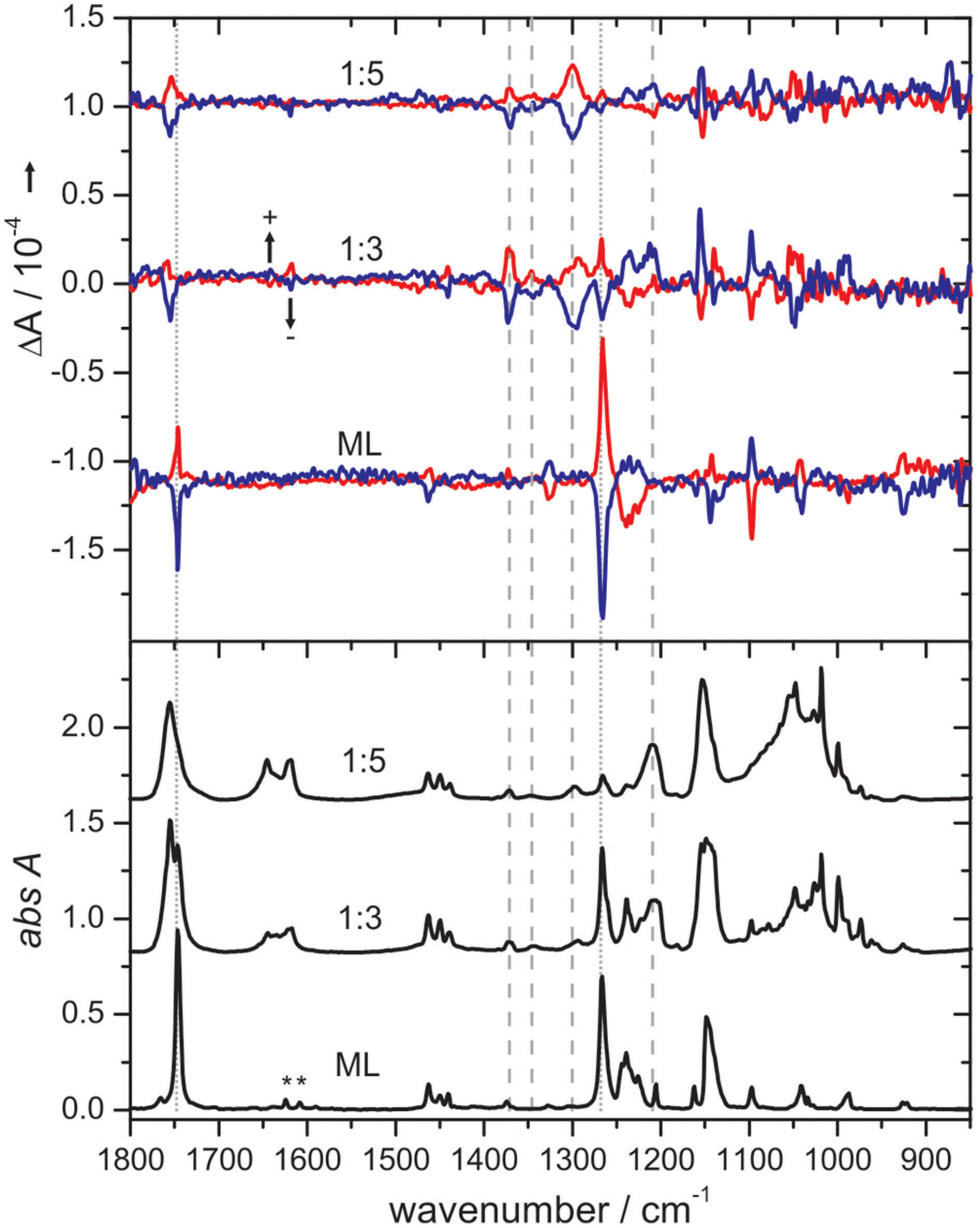
**Comparison of IR and VCD spectra of pure ML (obtained at 10 K) with those obtained for the 1:3 and 1:5 ML/NH_**3**_ mixtures at 30 K in cold Ar matrices**. The bands of residual water in the matrix are marked with an asterisk. The dotted and dashed lines indicate characteristic bands of the ML monomer and the ML-NH_3_ complex, respectively, whose intensities change with the sample composition. Reproduced with permission from Merten and Xu (2013c). Copyright 2013, Wiley-VCH Verlag GmbH & Co. KGaA, Weinheim.

To further confirm that such small H-bonded complexes generate the VCD features observed in solution, Merten and Xu carried out the matrix isolation VCD spectroscopic studies of the H-bonded chiral complexes prepared in cold rare gas matrices (Merten and Xu, 2013a,b,c). One example is the ML-NH_3_ complex whose IR and VCD spectra are shown in (Figure 9; Merten and Xu, 2013a,b). It is interesting to note that depositions at 10 K with different ML/NH_3_ ratios show drastic changes in the IR spectra but not in the VCD spectra, suggesting little formation of the chiral ML-NH_3_ complex but different NH_3_ clusters. On the other hand, depositions at 30 K yielded significantly different spectral patterns in both the IR and VCD spectra. At the 1:3 sample ratio, the ML monomer and the ML-NH_3_ complex coexist, whereas at the 1:5 ratio, the H-bonded complex becomes the dominant species. Two conformers of ML-NH_3_ were identified as the carriers of the observed IR and VCD spectra. We further note that the most stable conformer identified in this VCD study is the same one identified in the subsequent FTMW spectroscopic study (Borho and Xu, 2007; Thomas et al., 2013a,b).

**Figure 10:**
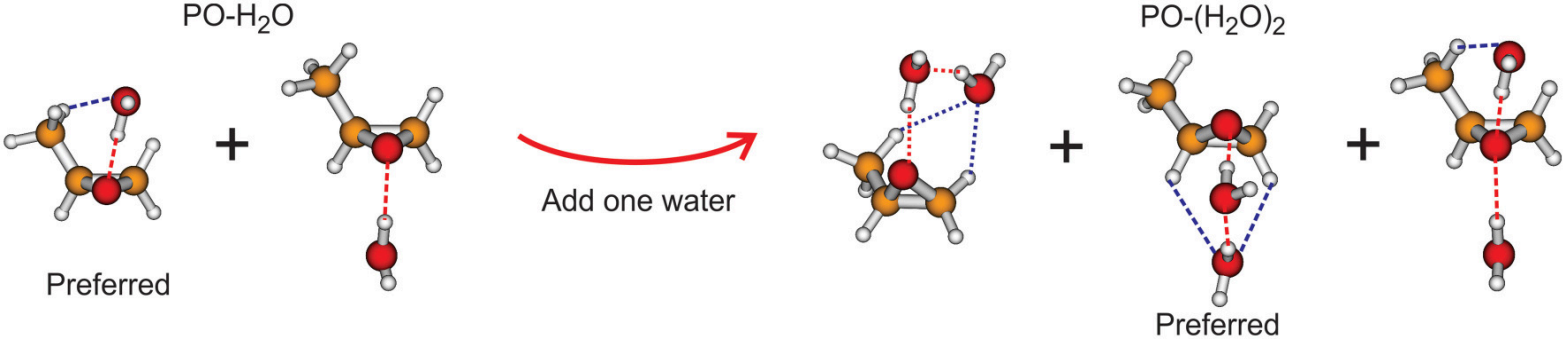
**Geometries of the two and three most stable conformers of PO-(H_**2**_O)_**1,2**_ obtained at the MP2/6-311++G(d,p) level of theory, respectively**. For the 1:1 Po-water complex, *syn* PO-water is preferred and for the 1:2 PO-(water)_2_ complex, *anti* PO-(water)_2_ is the preferred conformation (Su et al., 2006; Su and Xu, 2007a,b).

The situation with PO is a little different. Two distinct structural conformers, *syn* PO-H_2_O and *anti* PO-H_2_O were observed experimentally using FTMW spectroscopy (Su et al., 2006). In the gas phase, *syn* PO-H_2_O was found to be more stable than *anti* PO-H_2_O (Figure 10). The additional stability of the *syn* conformer was attributed to a secondary H-bond formed between methyl CH and the O atom of water. This is interesting since *anti* PO-H_2_O was the preferred conformation identified in solution rather than *syn* PO-H_2_O. In the subsequent study of the 1:2 complex (Su and Xu, 2007a,b), only *syn* PO-(H_2_O)_2_ and *anti* PO-(H_2_O)_2_ were detected experimentally, while the least stable conformer *bi* PO-(H_2_O)_2_ was not abundant enough to be observed under the condition of a supersonic jet expansion. In contrast to the 1:1 PO-H_2_O complex, *anti* PO-(H_2_O)_2_ is favored over *syn* PO-(H_2_O)_2_ (Figure 10). The possible explanation is that it is easier to optimize the interactions among two water molecules and PO without the interference of the methyl group in the *anti* configuration. The study demonstrates how conformational preferences may alter in the first few steps of solvation. It is interesting to note that the preference for *anti* vs. *syn* in the 1:2 hydration cluster is consistent with the observation of preference of *anti* in aqueous solution (Mukhopadhyay et al., 2006; Losada et al., 2008a). One may therefore hypothesize that interaction of the 1:1 PO-H_2_O cluster with the bulk water surrounding may be responsible for the switch of the conformational preference from the gas phase to aqueous solution. This leads naturally to the discussion about the importance of the bulk solvent environment.

### The importance of bulk solvent surrounding

**Figure 11:**
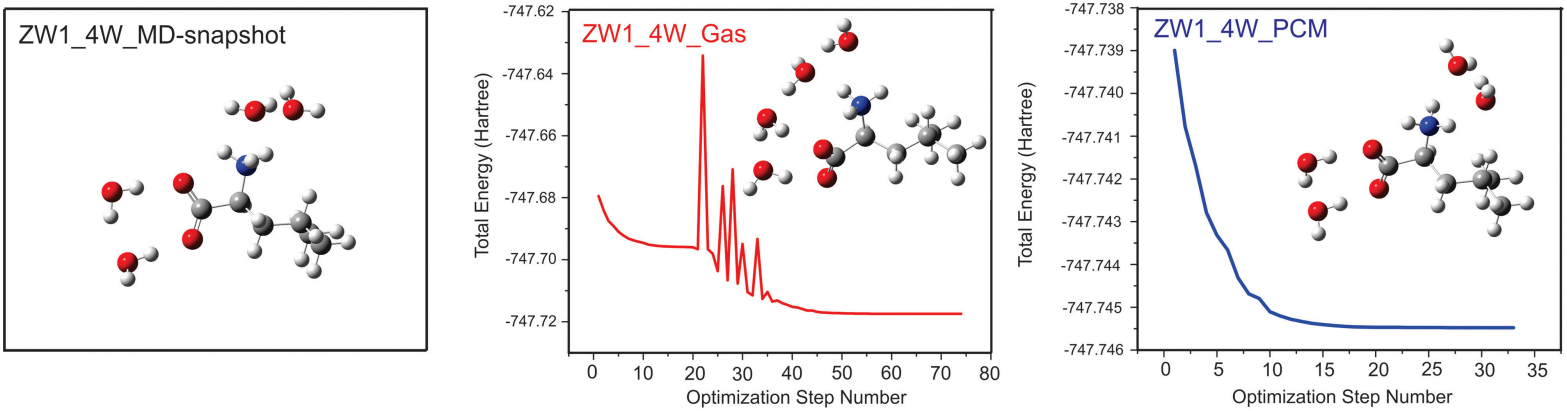
**Geometry optimizations carried out for the leucine ZW1-4W in the gas phase and in the presence of PCM of bulk water**. The initial MD snapshot is shown on the left. Reproduced with permission from Poopari et al. (2012b). Copyright 2012, American Institute of Physics.

**Figure 12:**
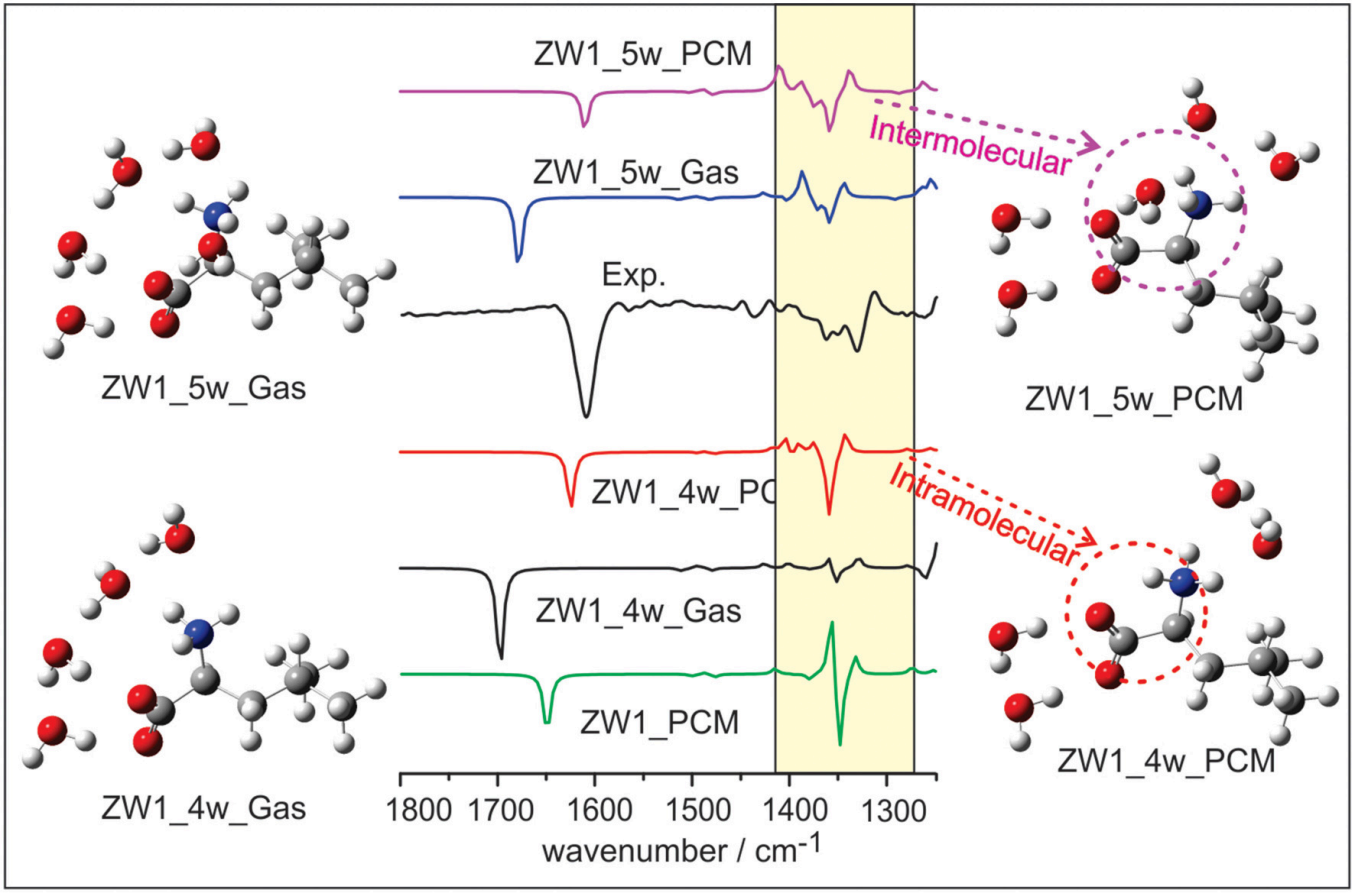
**The comparison of experimental VCD spectrum of leucine in water with the calculated ones of the leucine ZW1-5W and ZW1-4W complexes in the gas phase and in the PCM of water**. Reproduced with permission from Poopari et al. (2012b). Copyright 2012, American Institute of Physics.

Several VCD solvation studies of amino acids and their derivatives under different pH conditions have been reported recently (Zhu et al., 2012; Poopari et al., 2012a, 2013b). In these studies, it became quite clear that one must also take the bulk water solvent environment into account in order to reproduce the experimentally observed IR and VCD spectra. One amino acid studied under different pH condition is leucine (Poopari et al., 2012b). Leucine exists in three dominant species, i.e., the protonated, zwitterionic, and deprotonated forms, under three representative pH conditions of 1, 6, and 13, respectively. Through, the analysis of radial distribution functions around the O atoms of the COO^−^ group and H atoms of the ${\text{NH}}_{3}^{+}$ group, leucine hydration clusters with 4 and 5 water molecules are used to model the explicit H-bonding interaction between the leucine zwitterion and water. A representative snapshot of a hydration cluster consisting of a leucine zwitterion (ZW1) with 4 water molecules from the MD simulation is shown in Figure 11, as well as the geometry optimizations of the hydration cluster in the gas phase and in the PCM of bulk water solvent. The geometry optimization in the gas phase showed much variation in the process and resulted in a noticeably different H-bonded topology between water and ZW1. It appears that water molecules work hard to optimize the cooperative H-bonding interaction among themselves in the gas phase. The optimization done in the PCM of bulk water, on the other hand, converged quickly to a geometry similar to the representative MD snapshot, keeping the COO^−^ and ${\text{NH}}_{3}^{+}$ groups solvated. Clearly, the inclusion of the PCM of bulk water in the geometry optimization of the hydrates is important. In Figure 12, the experimental VCD spectrum is compared to the simulated VCD spectra of two hydration leucine clusters in the gas phase and in the PCM of water. As one can see, the experimental VCD spectrum is well reproduced by the simulated VCD features of the hydration cluster consisting of the leucine zwitterion and 5 water molecules in the PCM of water.

### ROA spectroscopy of chiral molecules in water

In recent years, ROA spectroscopy has been applied to solvation studies of amino acids and carbohydrates (Cheeseman et al., 2011; Hopmann et al., 2011; Kamiński et al., 2012). As in the case of VCD studies, the importance of considering both implicit and explicit water solvent effects was illustrated in the Raman and ROA studies of lactamide and 2-aminopropanol (Hopmann et al., 2011). The Raman and ROA spectra of lactamide and 2-aminopropanol were simulated using four different approaches. In the first approach, only the PCM model was used to account for the bulk water effects. In the second approach called “*ad hoc* hydration” by the authors, small hydration clusters of lactamide and 2-aminopropanol were placed in the PCM of bulk water to account for both implicit and explicit solvation effects. In the third and fourth approaches, classical MD and Car-Parrinello MD (CPMD) were used, respectively, for lactamide and 2-aminopropanol. These authors noted that the presence of explicit water molecules was required to provide better agreement with experimental spectra, including the inhomogeneous broadening of spectral bands, and finer intensity changes caused by solvation. This was achieved by “*ad hoc* hydration” or by using a large number of snapshots generated through either classical MD or CPMD. About 1000–2000 snapshots from the MD simulations were needed in order to achieve good agreement with the experimental data. A related test using the same approach with glycine, an achiral molecule, showed that the ROA spectrum simulated for glycine is reduced to zero *only* when more than 500 snapshots were used. It should be pointed out that these authors carried out only *partial* geometry optimizations of the snapshots in order to preserve the MD geometries of water molecules. Basically, the low-frequency modes corresponding to motion of water molecules were not optimized by using the normal-mode optimization procedure developed by Bouř and Keiderling (2002) and Hudecová et al. (2012).

In a subsequent Raman and ROA spectroscopic study of cysteine reported by some of the same authors, the microsolvation approach, i.e., placing small cysteine-(water)_4_ clusters in the PCM of bulk water, demonstrated the best agreement to the experimental Raman and ROA features (Kamiński et al., 2012). On the other hand, the simulated Raman and ROA spectral features using the similar classical MD approach as above do not agree well with the corresponding experimental data. It was noted that the MD conformational distribution differs very much from the ab initio results. The authors attributed this failure to the fact that the force field in AMBER02 is parameterized for the potential energy surface of an amino acid moiety in a protein, rather than for a free molecule (Kamiński et al., 2012).

In a follow-up study of ECD and ORD of lactamide and 2-aminopropanol again by some of the same authors (Pikulska et al., 2013), it was found that 200–300 snapshots are sufficient for the simulations of ECD spectra, in contrast to the simulations of the ROA spectra discussed above (Hopmann et al., 2011). Furthermore, it was pointed out that while the number of snapshots does not change the shape of the spectra significantly, the number of water molecules in the MD clusters does, especially the inclusion of the second solvation shell. The authors stated in their conclusion that “overall, no consistent improvement of the results over the microsolvation method or even PCM is observed when MD is used, which may result both from inadequacy of the employed force field (designed primarily for biopolymers) and of the exchange correlation functional.”

**Figure 13:**
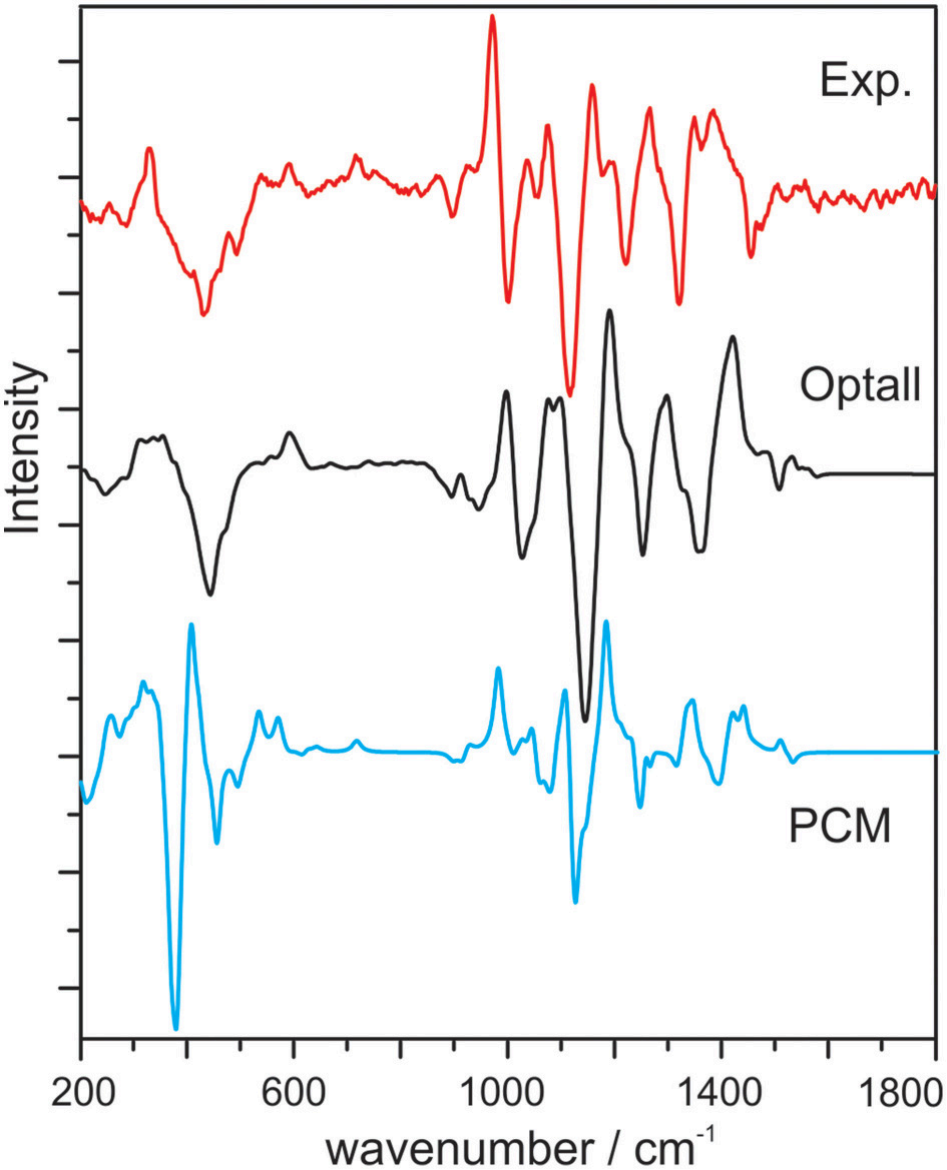
**Experimental ROA spectrum of methyl-β-D-glucose, calculated ROA spectrum with the optall process (see text for details), and calculated ROA spectrum using PCM**. All calculated spectra are 60:40 weighted averages of the gg and gt conformers of methyl-β-D-glucose. Reproduced with permission from Cheeseman et al. (2011). Copyright 2011, American Chemical Society.

Cheeseman et al. reported classical MD and ROA studies of methyl-β-D-glucose in water (Cheeseman et al., 2011). In this study, methyl-β-D-glucose is surrounded by two-layer explicit water molecules. The spectral simulation was carried out using the ONIOM method where methyl-β-D-glucose is treated quantum mechanically and the water molecules with MM. Initial geometries were obtained from snapshots of the MD simulation. In addition, the authors utilized an electronic embedding scheme which incorporates the partial charges of the MM region into the QM Hamiltonian. Two geometry optimization schemes were used: the optglc and optall models. In the optglc model, all water molecules were fixed in their positions, whereas in the optall model, the positions of the water molecules were also relaxed in addition to the chiral solute. Overall, the simulated Raman and ROA spectra using the optall scheme give the best agreement with the experimental data (Figure 13), while the optglc model gives similar results. Especially in the 200–500 cm^−1^, the MD simulation demonstrated superior performance over PCM in capturing the interactions between the aqueous environment and the monosaccharide. It is also interesting to note that only 16 snapshots were used for each sugar conformer to generate the final Raman and ROA spectra. This is a much smaller number of snapshots required compared to other studies reported (Hopmann et al., 2011; Kamiński et al., 2012), a point which will be further addressed in the next section.

### Clusters-in-a-liquid approach

Based on the combined experimental and theoretical vibrational optical activity studies presented, it is clear that one must consider both the implicit and explicit interactions with water in order to capture the water solvent effects adequately. We advocate a “clusters-in-a-liquid” model as an effective approach to achieve this goal. In this model, one builds the necessary explicit H-bonded chiral solute-(water)_n_ clusters, and then places the clusters in an implicit solvent model, for example PCM, or a more sophisticated ONIOM model of MM water molecules (Cheeseman et al., 2011) (vide infra), to account for the surrounding bulk environment. We would like to emphasize that in our model, the construction of the explicit H-bonded chiral solute-(water)_n_ cluster is based on the experimental pieces of evidence that there are such dominant chiral hydration species which are mainly responsible for the observed chiroptical spectral features, rather than the chiral solutes themselves. The main purpose in constructing the hydration clusters is to identify the dominant species in aqueous solution. This is different from MD cluster snapshot approach pursued by others. This is also somewhat different from the approach of using microsolvation clusters to capture many different H-bonding topologies between water and a chiral solute (Luber and Reiher, 2009). In some studies, the authors refer to the microsolvation clusters they built as *ad hoc* explicit hydration, implying some randomness in the cluster selection, especially since they recognized that choosing a different number of water molecules often resulted in different spectra (Hopmann et al., 2011; Kamiński et al., 2012; Pikulska et al., 2013). Generally speaking, such observations are consistent with our proposal that only some specific hydration clusters are the dominant species. For example, in the case of PO in water, only the 1:1 PO-water complex is the main species identified but not the 1:2 or 1:3 complexes (Losada et al., 2008a).

One critical question is how to build these important chiral hydration clusters which capture the most important explicit solute-water H-bonding interactions in aqueous solution. We have utilized two approaches. One approach is to apply MD simulations. For example, we have used Amber program^1^ with Amber ff99 force field and a box of TIP3P (Jorgensen and Jenson, 1998) or TIP4P (Jorgensen et al., 1983) water to solvate the targeted chiral solute. Then we analyzed the radial distribution functions centered at the potential H-bond donor and acceptor sites of the chiral solute to provide an estimate of the number of water molecules explicitly H-bonded to the solute at each site. Based on such estimate, the representative MD snapshots were selected and used as starting points for subsequent *complete* geometry optimizations using DFT. A second approach is to construct such hydration clusters based on one's chemical intuition and spectroscopic experience. For example, by examining the experimental IR spectrum, one may recognize the usual vibrational band shift for some characteristic IR bands due to H-bonding interactions with water and use such information to make an educated guess about the number and/or positions of water molecules. In our own dealing with water solvation, we recognize that often only the very tightly bound water molecules to a chiral solute are important. Larger hydration clusters with extensive water H-bonding networks are typically not the dominant species in aqueous solution, i.e., not the main carriers of the chiroptical signatures observed.

In an early study, Luber and Reiher (2009) found that explicit hydration of 1,6-anhydro-β-D-glucopyranose by a small number of water molecules led to very large changes in both Raman and ROA spectra. This is consistent with the observation shown in Figures 6, 8 where even very subtle change in the position of the water molecule, for example in the *insertion* 1:1 ML-water complex, can lead to opposite signs for some VCD bands. Such high sensitivity to the number of water molecules and their positions is likely to be one main reason why several thousand snapshots are needed in the previous MD studies to achieve ROA convergence (Hopmann et al., 2011; Kamiński et al., 2012). Many hydration clusters which are not the dominant species in such aqueous solution were included in the snapshots. Those trivial hydration clusters generate a large amount of extra ROA signatures and require a great deal of averaging to converge. Indeed, the test with glycine, an achiral molecule, indicates that more than 500 snapshots were needed to obtain the expected zero ROA spectrum (Hopmann et al., 2011). Since VCD and ROA are highly sensitive to subtle water position differences, for example non-bonded OH pointing directions, it is unclear if the current MD simulations or partial geometry optimizations of the snapshots can capture the subtle conformational preference faithfully. In the MD study of cysteine, the authors did note that the MD conformational distribution differs very much from the ab initio results (Kamiński et al., 2012).

Why did Cheeseman's study of methyl-β-D-glucose in water require only 16 snapshots? The particular study did not take the explicit H-bonding interactions between water and glucose into account at the quantum mechanics level and no Raman or ROA bands associated with water show up in the predicted spectra. Essentially, this study considered the effects of the explicit water molecules on the conformation of methyl-β-D-glucose and on ROA sign and intensity without the explicit H-bonds with water. One may consider this approach as a more sophisticated way to treat the bulk water environment than PCM. Indeed, in our “clusters-in-a-liquid” model, we advocate not to use explicit H-bonded chiral hydration clusters to account for solvent fluctuation for the reasons discussed above. Such fluctuation is better treated with a similar approach as Cheeseman et al. (2011), although more studies are needed to examine this issue in greater details. We emphasize the difference between the water molecules which are H-bonded to a chiral solute to form relatively long-lived hydration clusters and those surrounding the hydration clusters.

**Figure 14:**
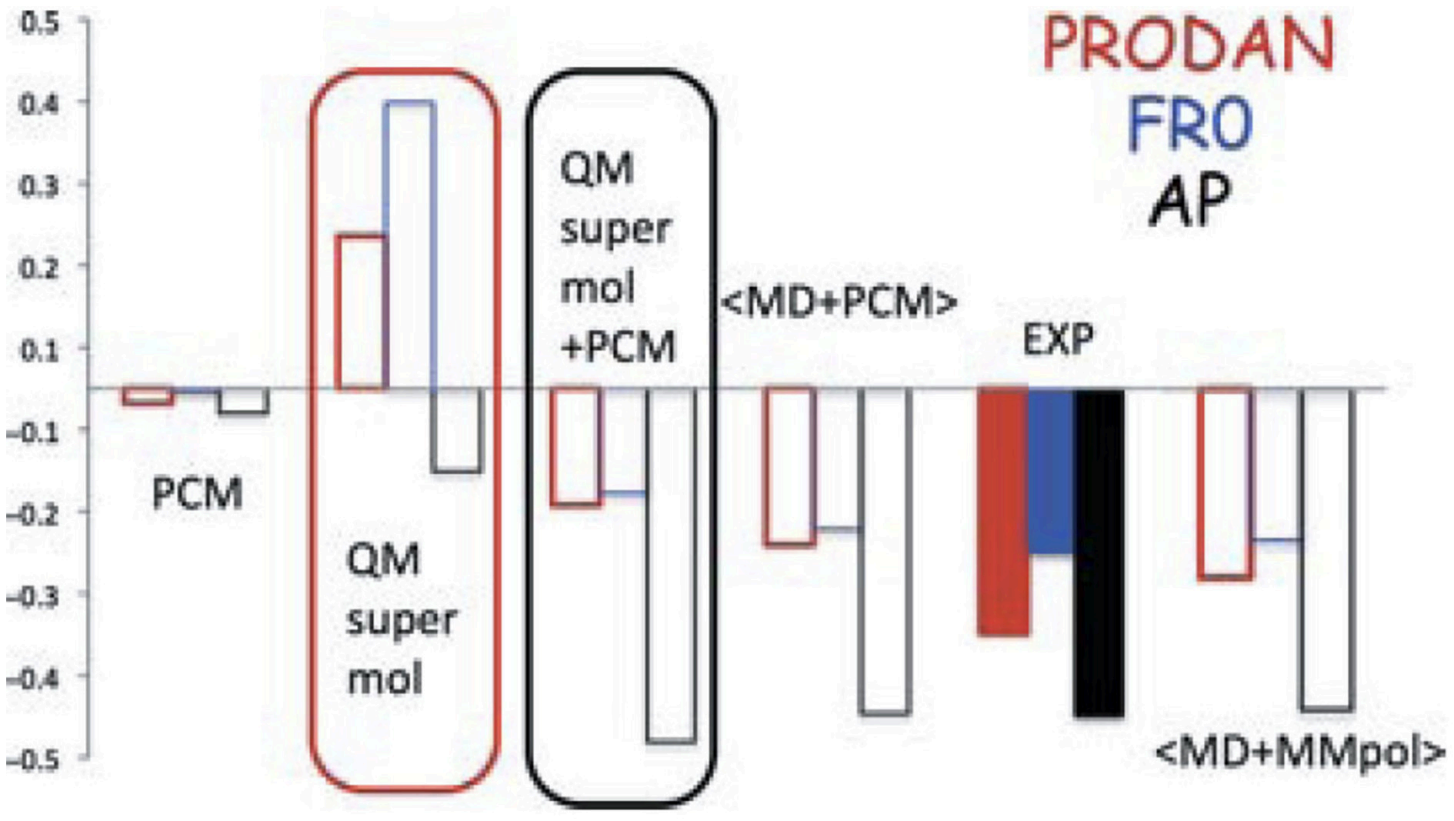
**TDCAMB3LYP/6-311+G(d,p)/PCM results of shifts (in eV) of the emission energies of AP, PRODAN, and FR0 moving from a polar (acetonitrile for AP and dimethylsulfoxide for PRODAN and FR0) to a protic polar (water for AP and PRODAN, methanol for FR0) solvent**. Reproduced with permission from Mennucci (2012). Copyright 2012, John Wiley & Sons, Ltd.

The “clusters-in-a-liquid” approach can also be utilized for other protic solvents (Debie et al., 2008; Dezhahang et al., 2013; Poopari et al., 2013a) and for other properties of molecules in protic solvents (Mennucci, 2012). In a recent review of PCM, Mennuci compared the performances of five different theoretical approaches in predicting the shifts of the emission energies of three typical fluorescent probes (Figure 14). Here, the solvatochromism (on both absorption and fluorescence) of three typical fluorescent probes, namely, 4-aminophthalimide (AP), 6-propionyl-2- dimethylamino naphthalene (PRODAN), and its recently synthesized analog 7-diethylamino-9,9-dimethyl-9H-fluorene-2-carbaldehyde (FR0) were calculated (Mennucci et al., 2010). As one can see, the combined explicit and implicit approach yielded excellent agreement with the experiment, whereas PCM or hydration clusters alone failed to capture the correct shift.

## Concluding remarks and future outlook

In this review, we have discussed the current approaches commonly used to account for water solvent effects in VCD and ROA spectra obtained in aqueous solution. It is generally acknowledged that both the explicit H-bonding interactions between a chiral solute and water molecules and the implicit water bulk environment are important. In practice, how these two solvent effects are modeled differ depending on the specific methods chosen. Experimental VCD spectra of some prototype chiral compounds, such as PO, ML, and amino acids, especially the chirality transfer spectral features in the water bending band region, together with the related theoretical simulations, support the following view. Some small chiral hydration clusters, rather than the chiral solutes themselves, are the dominant species in aqueous solution and are the ones that contribute mainly to the experimentally observed VCD features. This conclusion has also been supported by the related ORD studies and also rotational spectroscopic studies of the related small hydration clusters, as well as the matrix isolation VCD studies of small H-bonded chiral clusters. Based on this experimental and theoretical foundation, we advocate the “clusters-in-a-liquid” approach as an effective way to account for water solvent effects in chiroptical measurements. That is to identify the main species in aqueous solution by constructing some small chiral hydration clusters in the bulk water environment modeled by, for example, PCM. We have discussed some methods used to find these representative hydration clusters. We emphasize that the success of the “*ad hoc* hydration” is not random but rather rooted in this general view that some small hydration clusters are responsible for the chiroptical signatures observed. We have also analyzed the differences between this approach and the MD snapshot approach and offered some explanations about the difficulties one faces in the MD snapshot averaging approach.

Because of the complexity of condense phase, it is often challenging to prove a point of view beyond any doubts. To strengthen the support for the “clusters-in-a-liquid” approach, future work involving simulations of both VCD and ROA spectra of a chiral compound in solution would be desirable. Since one would expect the same dominant species to be responsible for both VCD and ROA spectra, such studies would apply an even more stringent test of this approach. In addition, future work in matrix isolation VCD spectroscopy of sequential solvation of a prototype chiral compound with several water molecules would be highly valuable. One may anticipate the observation of some strong chirality transfer spectral features which are not detected in aqueous solution, thus experimentally verifying that some cluster sizes are not the dominant species in aqueous solution. Conformer specific rotational spectroscopic studies of sequential solvation of a chiral compound allow the clearly identification of subtle conformational differences and also provide the comparison to those identified in the gas phase or matrix isolation and in solution VCD studies. Compared to ROA, VCD spectral features are even more sensitive to subtle conformational differences such as the non-bonded OH pointing directions which may be difficult to capture correctly in a MD simulation. Future MD studies including *both* VCD and ROA simulations in aqueous solution will be important to further evaluate the MD snapshot approach. While the PCM method has been used conveniently and largely successfully in the “clusters-in-a-liquid” approach to reproduce the effects of the bulk water environment, it would be desirable to explore the utility of other models, for example the two-layer ONIOM model utilized by Cheeseman et al. in the glucose study. The representation of bulk water with explicit MM water molecules rather than a structureless continuum characterized by its dielectric constant may capture some new spectral features which were missed with PCM.

## Author contributions

AP wrote the first draft on the solution VCD and ROA studies. JT wrote some part of the gas phase studies. MP was involved in discussing and refining the article. YX wrote the main portion of the review article and edited and finished the current version.

## Funding

This research was funded by the University of Alberta, the Natural Sciences and Engineering Research Council of Canada, the Canada Foundation for Innovation, and the Alberta Enterprise and Advanced Education. We also gratefully acknowledge access to the computing facilities of the Shared Hierarchical Academic Research Computing Network (SHARCNET: www.sharcnet.ca), the Western Canada Research Grid (Westgrid), and Compute/Calcul Canada. YX holds a senior Canada Research Chair in Chirality and Chirality Recognition.

### Conflict of interest statement

The authors declare that the research was conducted in the absence of any commercial or financial relationships that could be construed as a potential conflict of interest.

## Footnotes

^1^Baerends, E. J., Ziegler, T., Autschbach, J., Bashford, D., Bérces, A., Bickelhaupt, F. M., et al. *Amsterdam Density Functional Program*. Vrije Universiteit: Amsterdam. Avaliable online at: http://www.scm.com
